# A rare case of Burkitt's lymphoma of the breast in a 19‐year‐old male: Case report

**DOI:** 10.1002/ccr3.8081

**Published:** 2023-10-17

**Authors:** Ronald Kato, Kibudde Solomon

**Affiliations:** ^1^ Department of Emergency Medicine Savannah Hospital Nairobi Kenya; ^2^ Division of Radiation Oncology Uganda Cancer Institute Kampala Uganda

**Keywords:** biopsy, Burkitt lymphoma, histology, non‐Hodgkin's lymphoma, tumor

## Abstract

**Key Clinical Message:**

Burkitt's lymphoma is a highly aggressive non‐Hodgkin's lymphoma that can affect various parts of the body, which include jaw, facial bones, retroperitoneum, and abdominal viscera, it is worth noting that breast involvement is extremely rare in Burkitt's lymphoma and has not been documented.

**Abstract:**

Burkitt's lymphoma is a highly aggressive non‐Hodgkin's lymphoma that can affect various parts of the body including breast. This sarcoma is identified as a rapidly fatal malignant lymphoma syndrome and 50% of all malignant tumors in children. However, breast involvement has not been documented. A 19‐year‐old male presented to our emergency department with a left breast swelling for 2 months associated with drenching night sweats, weight loss and evening fevers. Physical examination revealed a mass measuring approximately 15 × 16 × 15 cm in the widest dimension, skin hyperpigmentation, no nipple discolorations, discharges, and non‐tender on palpation. Biopsy was done, and the histology report revealed sheets of monomorphic medium lymphocytes with a high mitotic rate and frequent apoptotic bodies showing a starry‐sky appearance. The immunohistochemistry report revealed positive staining for Ki‐67, CD‐20, and CD‐10 tumor markers and CD45 on flow cytometry. The patient was started on aggressive hydration, rasubricase administration, CODOX‐M/IVAC regimen 6 cycles, and G‐CSF and registered significant reduction in the size of the mass. Burkitt's lymphoma is a highly aggressive non‐Hodgkin's lymphoma that can affect various parts of the body. It commonly involves the jaw, facial bones, retroperitoneum, and abdominal viscera. The disease typically affects young patients in areas of high incidence, such as the jaw, whereas visceral involvement is more common in older patients in low‐incidence areas. It is worth noting that breast involvement is extremely rare in Burkitt's lymphoma.

## Graphical abstract

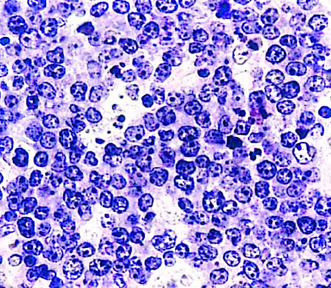

## 1 INTRODUCTION

Burkitt's lymphoma is a highly aggressive non‐Hodgkin's lymphoma that can affect various parts of the body including breast. This sarcoma is identified as a rapidly fatal malignant lymphoma syndrome and 50% of all malignant tumors in children. However, breast involvement has not been reported up to date.^1^

Involvement of the jaw and facial bones, retroperitoneum, abdominal viscera, ovaries, spinal epidural, and bone marrow has been described; however, jaw involvement is usually seen in younger patients in endemic areas of high incidence, while visceral involvement is noted more commonly in older patients in areas of low incidence.^2^ It is worth noting that breast involvement is extremely rare in Burkitt's lymphoma.

## 2 CASE REPORT

**FIGURE 1:**
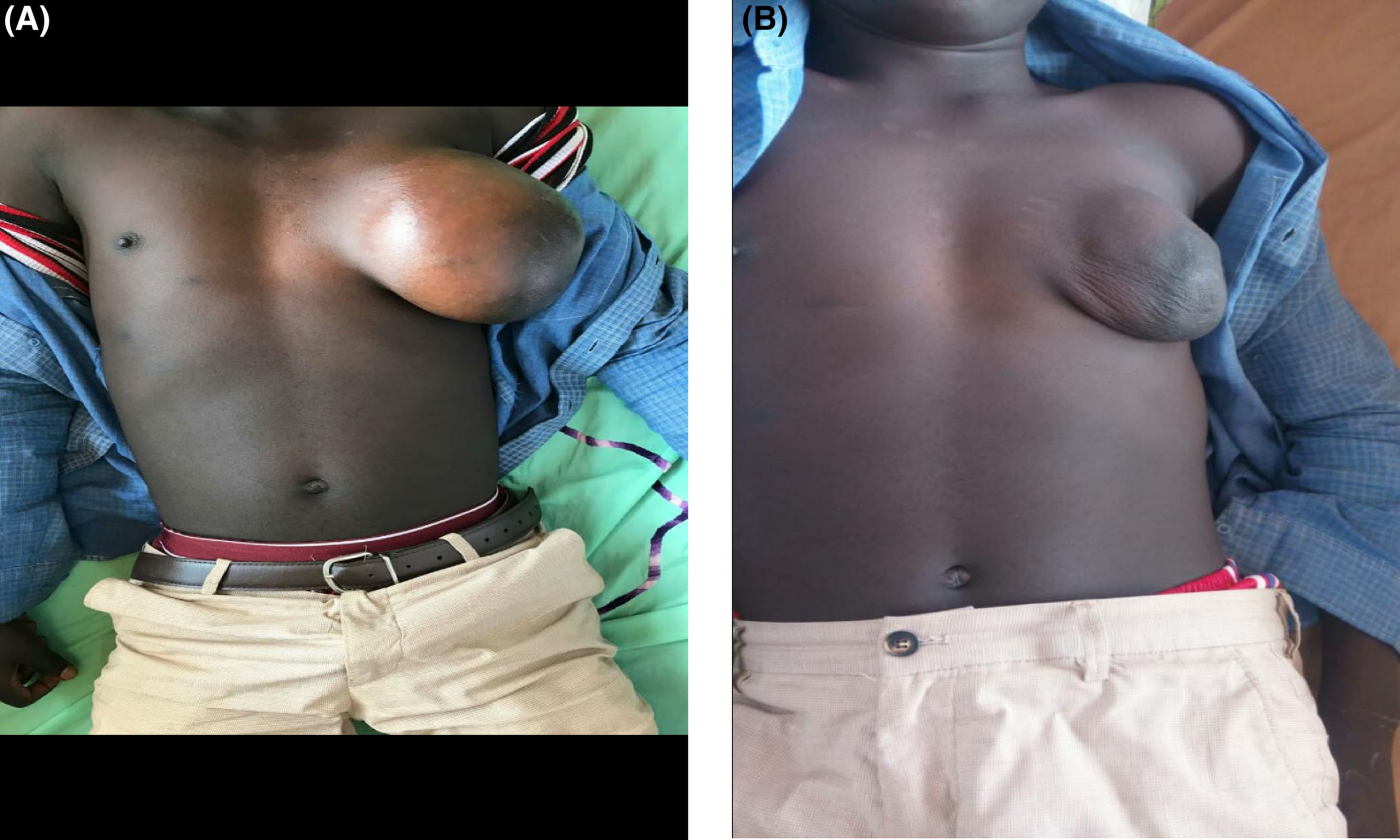
(A) Showing the breast mass before chemotherapy.(B) showing the breast massreduced significantly after 6th cycle of chemotherapy.

A 19‐year‐old male presented to our emergency department with left breast swelling for 2 months with drenching night sweats, weight loss, and evening fevers. A physical examination revealed a mass measuring approximately 15 × 16 × 15 cm in the widest dimension, skin hyperpigmentation, no discharge from the nipples, no nipple skin discoloration, and non‐tender on palpation. There were also palpable, non‐tender axillary lymph nodes. The patient was admitted in our emergency department clerked and done the necessary investigations which included the biopsy of the breast mass. (Figure 1A,B).

The patient underwent a pre‐chemotherapy workup that included a lumber puncture for cerebral spinal fluid analysis to rule out central nervous system involvement, For CSF, opening pressure was 18cmH_2_O(6–25cmH20), glucose: 2.7 mmol/L(2.5–4.4 mmol/L), and total protein: 30 mg/dL.(15–60 mg/dL.) WBC: 4.0/ul(<5.0/ul). Serum LDH: 25 U/L(<40.0 U/L), renal function tests: urea 6.0 mmol/L(2.5–8 mmol/L), creatinine: 70 mmol/L, (63–115 mmol/L), potassium: 4.0 meq/L(3.5–5.5 meq/L), sodium:136 meq/L(135–155 meq/L), calcium: 6.0 mg/dL (8.5–10.5 mg/dL), complete white blood cell count 5.0 (4.0–10.0 × 10^9^/L), chest CT‐scan was done and showed no pulmonary metastases, PET scan and bone marrow biopsy and aspirate were not done due to financial constraints.

**FIGURE 2:**
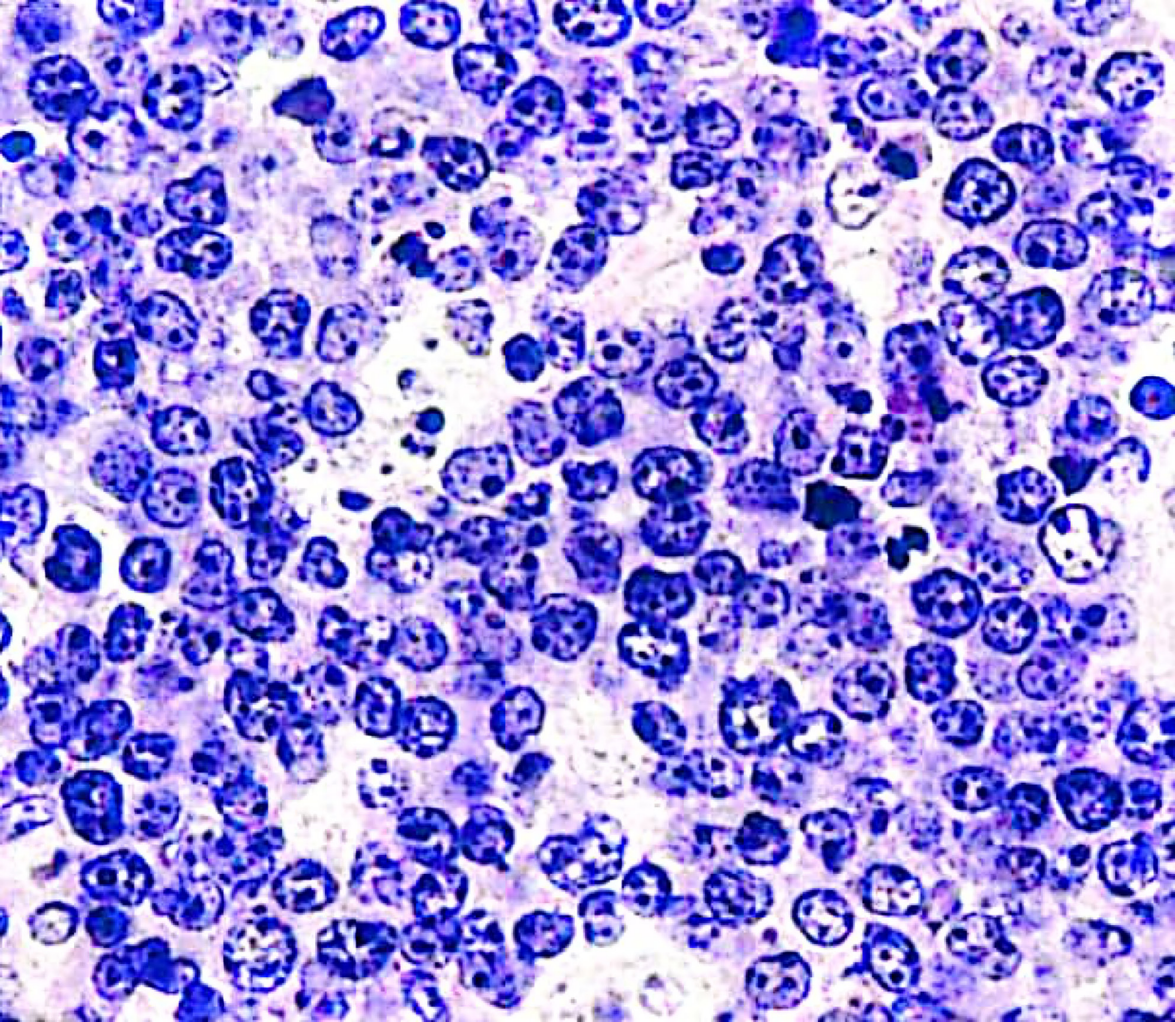
H&E stained section. The cells show cytoplasmic borders with molded appearance and proliferation with increased mitotic activity and increased apoptosis sheets of monomorphic medium lymphocytes showing a starry‐sky appearance.

Biopsy of the breast mass was done, and on hematoxylin–eosin stained section, the cells had cytoplasmic borders giving them a molded appearance and highly proliferative, which was reflected in the increased number of mitotic figures and Increased cell turnover with increased apoptosis sheets of monomorphic medium lymphocytes showing a starry‐sky appearance. (Figure 2).

**FIGURE 3:**
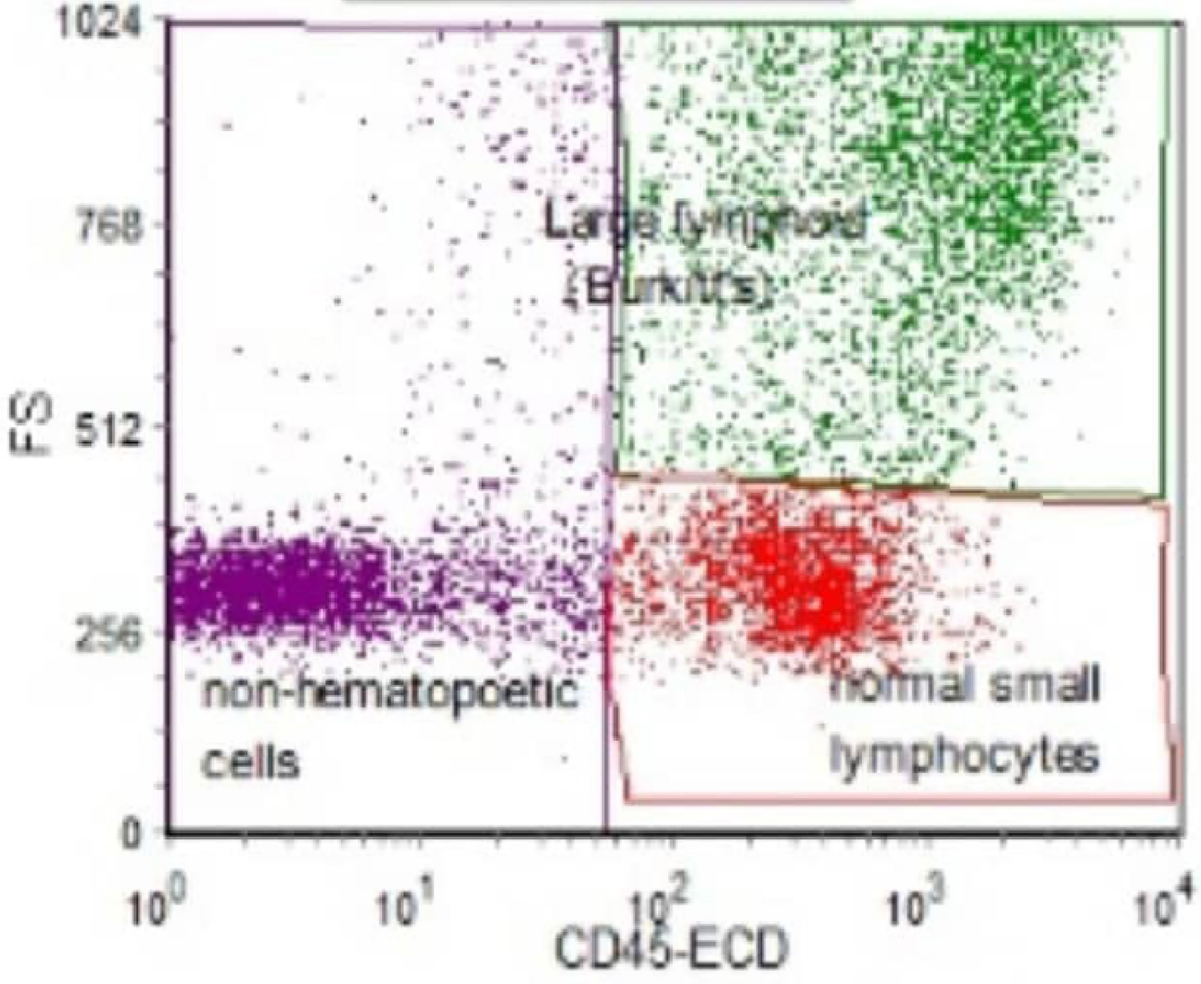
Cells identified using a SSC versus CD45plot with the cells show cytoplasmic borders with moderate to high SSC/FSC and bright CD45.

The immunohistochemistry report revealed positive staining for Ki‐67, CD‐20, and CD‐10 tumor markers and CD45 on flow cytometry (Figure 3). The CODOX‐M/IVAC regimen was the chemotherapy protocol that was used based on its success rate.

This form of combination has been found to be more effective than using any drugs alone. In CODOX‐M/IVAC the drugs are cyclophosphamide, vincristine (Oncovin), doxorubicin, and Methotrexate. In IVAC regimen, the drugs are isocyanide, etoposide (Viperid), and cytarabine (known as Ara‐C), (Table 1).

**TABLE 1:** The treatment schedule was as shown above.

Cycle/course	Treatment
1	CODOX‐M
1	IVAC
2	CODOX‐M
2	IVAC

*Note*: One cycle of CODOX‐M, 1 cycle of IVAC and another 2 cycles of CODOX‐M, and 2 cycles of IVAC.

The drugs were delivered through a central venous access device (CVAD), and there were six cycles based on this regimen. In between the cycles, filgrastim (G‐CSF) was given to allow the blood count levels to recover. Once the blood count levels recovered, the next cycle was planned.

## 3 CASE DISCUSSION

Burkitt lymphoma can be divided into sporadic, endemic, and immunodeficiency‐related variants. In developed countries where sporadic cases are most commonly encountered, Burkitt lymphoma can occur at any age, although the peak incidence is in the first decade of life.

Burkitt lymphoma accounts for 30% of pediatric lymphomas and less than 1% of lymphoma cases in adults. The disease incidence is 2–3 times higher in equatorial Africa. Termed endemic Burkitt lymphomas, the majority of cases are Epstein–Barr virus (EBV)‐related. The immunodeficiency‐related variant is most commonly associated with the human immunodeficiency virus (HIV).^3^

Involvement of the jaw and facial bones has been described and seen usually in younger patients in endemic areas of high incidence, while visceral involvement is noted more commonly in older patients in areas of low incidence.^1^ It is worth noting that breast involvement is extremely rare.

The diagnosis is most readily made by an excisional lymph node biopsy; in cases where an excisional lymph node biopsy is difficult, multiple core needle biopsies may be done, and a hematopathology review is essential.^4^

On hematoxylin–eosin‐stained sections, the cells have cytoplasmic borders, giving them a molded appearance. The neoplastic cells are highly proliferative, which is reflected in the increased number of mitotic figures. Increased cell turnover is countered by increased apoptosis resulting in a distinct starry‐sky pattern of classic Burkitt lymphoma with histologic proliferative fraction approaching 100%. While more unusual variants may be harder to distinguish, the most commonly used measure of proliferation is the Ki‐67, where nearly 100% of the cells stain positive for Ki‐67, and most Burkitt lymphoma cells can be initially identified using a SSC versus CD45 flow cytometry plot. These cells have moderate to high SSC/FSC and bright CD45.^5^

In developing countries, the stage of the disease and abdominal ultrasound are used to determine the prognosis. Fluorodeoxyglucose positron emission tomography (PET) scans and CT scans are used to evaluate for residual disease.^6^

The rapid institution of chemotherapy, ideally within 48 h of presentation, is of utmost importance in Burkitt lymphoma. Aggressive hydration and recombinant urate oxidase (rasubricase) for hyperuricemia are currently recommended as prophylaxis against tumor lysis syndrome and to improve the patient's performance status.^7^

### 3.1 Other treatment regimens and long term survival rates

Based on experiences with other malignancies, patients with Burkitt lymphoma may experience a rapid improvement with treatment such as fractionated cyclophosphamide or a single cycle of CHOP chemotherapy, which can improve a patient's performance status. While most patients will initially respond to treatment using CODOX‐M/IVAC regimen, investigators have reported two‐year overall survival ranging from 67%–92%.

Three‐year overall survival for hyper‐CVAD has ranged from 49% to 89%. And six‐year overall survival for DA‐EPOCH‐R has been reported to be 100%. And for patients who relapse after initial therapy, salvage options should be considered palliative given few patients achieve long‐term survival.

Few options exist for patients who relapse after initial therapy. Typical salvage regimens used in diffuse large B‐cell lymphoma such as ICE (isocyanide, cytarabine, and etoposide) or DHAP (dexamethasone, cytarabine, and cisplatin) can sometimes be used.

### 3.2 Differential diagnoses

The differential diagnosis for adolescent breast masses is similar to those in adults and includes phyllodes tumors, primary breast cancer, sarcoma, lymphangioma or hemangioma, metastatic cancer, intraductal papilloma, fibroadenoma (and giant fibroadenoma), abscesses, and benign cysts.

### 3.3 Prognostic factors

For good prognosis includes isolated jaw mass and completely resectable mass, while bad prognosis includes male sex, intra‐abdominal mass, relapse, CNS involvement, and bone marrow involvement. Treatment of limited‐stage (stages I and II) Burkitt lymphomas is usually very successful, with a long‐term survival rate of over 90%. The long‐term survival rate for children and teens with more advanced (stage III or IV) Burkitt lymphoma ranges from about 80% to 90%.

## 4 CONCLUSION

Burkitt's lymphoma is a highly aggressive non‐Hodgkin's lymphoma that can affect various parts of the body. It commonly involves the jaw, facial bones, retroperitoneum, abdominal viscera, ovaries, spindle epidural, and bone marrow. The disease typically affects young patients in areas of high incidence, such as the jaw, whereas visceral involvement is more common in older patients in low‐incidence areas. It is worth noting that up to date breast involvement is extremely rare in Burkitt's lymphoma.

## AUTHOR CONTRIBUTIONS

**Ronald Kato:** Conceptualization; formal analysis; investigation; methodology; writing – original draft; writing – review and editing. **Kibudde Solomon:** Methodology; project administration; software; supervision; validation.

## FUNDING INFORMATION

There was no funding or any financial assistance that was contributed towards this manuscript.

## CONFLICT OF INTEREST STATEMENT

There is no conflict of interest between authors and all our institutions in regarding to publication of this report.

## CONSENT

A written informed consent was obtained from the patient to publish this report in accordance with the Journal's patient consent policy. From the patient, “***I confirm that, the case report has been fully explained to me and all my questions have been answered to my satisfaction, I therefore have no objection to the publication of this report***.”

## PATIENT PERCEPTION ABOUT THIS CASE

The patient's perception is that the swelling was something that might be related to witch craft from his village and also related it to cancer of the breast.

## Data Availability

The data that support the findings of this study are openly available; (1) https://www.annualreviews.org/doi/abs/10.1146/annurev‐immunol‐032414‐112326 (2) https://bjssjournals.onlinelibrary.wiley.com/doi/abs/10.1002/bjs.18004619704
